# Anatomical contribution of the orbicularis oculi to the zygomaticus major: An improved understanding of the smile with consideration for facial cosmetic procedures

**DOI:** 10.1371/journal.pone.0272060

**Published:** 2022-07-28

**Authors:** Joe Iwanaga, Mi-Sun Hur, Shogo Kikuta, Soichiro Ibaragi, Koichi Watanabe, R. Shane Tubbs

**Affiliations:** 1 Department of Neurosurgery, Tulane Center for Clinical Neurosciences, Tulane University School of Medicine, New Orleans, LA, United States of America; 2 Department of Neurology, Tulane Center for Clinical Neurosciences, Tulane University School of Medicine, New Orleans, LA, United States of America; 3 Dental and Oral Medical Center, Kurume University School of Medicine, Kurume, Fukuoka, Japan; 4 Division of Gross and Clinical Anatomy, Department of Anatomy, Kurume University School of Medicine, Kurume, Fukuoka, Japan; 5 Department of Oral and Maxillofacial Anatomy, Graduate School of Medical and Dental Sciences, Tokyo Medical and Dental University, Tokyo, Japan; 6 Department of Anatomy, Daegu Catholic University School of Medicine, Daegu, Korea; 7 Department of Oral and Maxillofacial Surgery, Okayama University Graduate School of Medicine, Dentistry and Pharmaceutical Sciences, Okayama, Japan; 8 Department of Anatomical Sciences, St. George’s University, St. George’s, Grenada, West Indies; 9 Department of Structural & Cellular Biology, Tulane University School of Medicine, New Orleans, LA, United States of America; 10 Department of Surgery, Tulane University School of Medicine, New Orleans, LA, United States of America; 11 Department of Neurosurgery and Ochsner Neuroscience Institute, Ochsner Health System, New Orleans, LA, United States of America; 12 University of Queensland, Brisbane, Australia; UNITED STATES

## Abstract

The aim of the present study was to determine the contribution of the orbicularis oculi (OOc) to the zygomaticus major (Zmj) in connecting the orbital region to the corner of the mouth. The OOc and Zmj of 22 embalmed adult Korean cadavers were dissected in 44 hemifaces. The OOc fibers were traced to determine their contribution to the Zmj. Parts of the superficial bundle in the orbital region of the OOc extended directly or indirectly to the Zmj in 22.7% of the specimens. The anatomical contribution of the OOc to the Zmj was divided into three categories depending on whether the connection between them was direct or indirect: (1) superficial orbital OOc fibers extended directly to the Zmj in 6.8% of the specimens, (2) superficial orbital OOc fibers extended to the zygomaticus minor and their small portion joined to the upper fibers of the Zmj in 15.9% of the specimens, and (3) no connection was identified between the OOc and Zmj in 77.3% of the specimens. The results of this study provide further anatomical insight into the relationship between the OOc and zygomaticus muscle complex. This information could be helpful for elucidating the anatomy of smiling and treatment and surgery designs for balanced or ideal smiles.

## Introduction

Mouth and orbital region movements are involved in several facial expressions such as different smile types. Smiles can also be classified into Duchenne (genuine) and non-Duchenne (nongenuine) [1, 2]. A Duchenne smile involves the combined actions of the orbicularis oculi (OOc), which raises the cheeks, and the zygomaticus major (Zmj), which pulls the lip corners, and is a true indicator of enjoyment [1–3]. A non-Duchenne smile only involves the Zmj [1, 2]. OOc contraction causes the distinctive crow’s feet wrinkles that represent a genuine smile [2].

Rubin et al. (1989) [4] and Manjula et al. (2015) [5] described a smile as being formed in stages: in the first stage, the levator muscles contract and raise the upper lip toward the nasolabial fold; and in the second stage, the levator labii superioris, Zmj, and buccinator muscles further raise the lips. The final stage is often characterized by squinting; it represents the periocular musculature contraction which supports maximum upper-lip elevation through the fold. These first and second stages seem to be associated with a non-Duchenne smile, and the final stage with a Duchenne smile.

Rubin (1974) [6] distinguished three types of smiles among 100 people based on the elevation and depression directions of the lip and the dominant muscle groups involved. In the “Mona Lisa” smile, which was observed in 67% of subjects, the corners of the mouth are pulled up and outwards, and the levators of the upper lips then contract to reveal the upper teeth; here the Zmj is dominant. In the “canine” smile (31%), the levator labii superioris is dominant and contract first, exposing the canine teeth. The corners of the mouth also contract to pull the lips upwards and outwards. In the “full denture” smile (2%), the elevators of the upper lips and the corners of the mouth, and the depressors of the lower lips all contract simultaneously, revealing the entirety of the upper and lower teeth. All muscles are equally dominant in this type of smile.

While the OOc and the zygomaticus minor (Zmi) have been reported to be anatomically connected [7, 8], the morphological features of the connection connections between the OOc and Zmj have not been elucidated. If this anatomical connection is present, its frequency, shape, strength, and other parameters would help to determine the anatomical relationship and coordination between the involved muscles.

The aim of this study was to determine the contribution of the OOc to the Zmj, which connects the orbital region and the corner of the mouth. The obtained data will help to explain one of the connected movement mechanism of the orbital and oral regions that induce smiles. They will also provide a reference for treatment and surgery designs for balanced or ideal smiles and for use in electromyographic studies.

## Materials and methods

This study examined the OOc and Zmj of 44 specimens from 22 embalmed adult Korean cadavers (10 males and 12 females) with a mean age of 72.1 years (range: 40–94 years) at the time of death. Their facial muscles were dissected to expose the OOc, Zmj, and Zmi. Their OOc fibers were traced to determine their contribution to the Zmj. No previous surgical scars or obvious pathologies were observed in the periorbital or perioral regions.

This study was approved by the Institutional Review Board of the Catholic Kwandong University (IRB no. CKU-21-01-1103). All cadavers had been legally donated to the Catholic Kwandong University College of Medicine. Donors voluntarily consented to the dissection and preservation of their body for education and research purpose in accordance with the act on dissection and preservation of corpses before their deaths. Families of donors fully agreed with the contents of the donor’s will to donate the body to the Catholic Kwandong University College of Medicine and promise to uphold that will. Details of body donors about cause of death and collection period are shown in Table 1. The study was performed in accordance with the requirements of the Declaration of Helsinki (64^th^ WMA General Assembly, Fortaleza, Brazil, October 2013).

**Table 1:** Details of body donors with regard to cause of death and collection period.

Number of cadavers	Sex	Cause of death	Collection period
1	Male	Coniosis	May, 2014
2	Female	Liver cirrhosis	June, 2014
3	Male	Cerebellar tumor	November, 2014
4	Female	Pneumonia	May, 2015
5	Female	Pneumonia	October, 2015
6	Male	Natural death	October, 2015
7	Male	Bladder cancer	March, 2016
8	Female	Ventricular fibrillation	May, 2016
9	Female	Lung cancer	June, 2016
10	Female	Cerebral infarction	September, 2016
11	Male	Pneumonia	October, 2016
12	Female	Common bile duct cancer	November, 2016
13	Male	Stomach cancer	November, 2016
14	Male	Pneumonia	November, 2016
15	Male	Hepatorenal syndrome	January, 2017
16	Female	Pancreatic cancer	June, 2017
17	Male	Renal failure	July, 2017
18	Female	Colorectal cancer	July, 2017
19	Female	Oral cancer	September, 2017
20	Female	Breast cancer	April, 2018
21	Female	Pneumonia	June, 2018
22	Male	Parkinson disease	July, 2018

## Results

Parts of the superficial bundle at the orbital region of the OOc extended directly or indirectly to the Zmj in 10 of the 44 hemifaces (22.7%, 6 from males and 4 from females). The anatomical contribution of the OOc to the Zmj was divided into the following three categories depending on whether their connection was direct or indirect:

- In Type I (three hemifaces, one male and two female, 6.8%; S1A Fig), the superficial bundle of the OOc extended directly to the Zmj and Zmi, constituting fibers of both. These extended fibers were connected in the medial and distal parts of the Zmj and Zmi, respectively. In this type the Zmi and Zmj had completely separate courses until they reached their insertion points.
- In Type II (seven hemifaces, five male and two female, 15.9%; S1B Fig), the superficial bundle of the OOc extended to the superficial bundle of the Zmi, and the inferior part of the superficial Zmi descended and merged with the upper fibers of the Zmj. The merging sites of the Zmi fibers that extended from the superficial OOc to the Zmj via the Zmi were in the medial or distal part of the Zmj. Before merging, the Zmi and Zmj ran close to each other, and after merging they ran separately some distance apart.
- In Type III, no connection was found between the OOc and Zmj in 34 hemifaces (77.3%, 16 males and 18 females; S1C Fig).

Type I had a more-distinct and thick fibers connecting the OOc and Zmj than Type II. The connecting fibers were symmetrically present in one female (Type I) and one male (Type II). The other Zmj fibers had normal origins and patterns in specimens that had a connection between the OOc and Zmj.

## Discussion

The results of this study indicate that anatomical connections between the OOc and Zmj are not common (22.7%), suggesting that these muscles contract independently during a Duchenne smile. In Type I, OOc contraction can further elevate the corner of the mouth laterally and upwards during smiling, and coordinate the movements of the mouth and orbital regions. It is thought that type I is likely to induce the “Mona Lisa” smile while type II is the “canine” smile.

### Hypothesis on Duchenne smiling

Russell and Fernández-Dols (1997) [1] reported that approximately one-half of Duchenne smiles were immediately preceded by non-Duchenne smiles, and approximately one-quarter of non-Duchenne smiles were immediately preceded by Duchenne smiles. OOc contraction is the difference between a non-Duchenne and a Duchenne smile.

Russell and Fernández-Dols (1997) [1] described potential sources of the coordination involved in Duchenne smiling, one of which was the physical effects of Zmj contraction on OOc contraction. As the Zmj lifts the corners of the mouth it raises the cheek, functioning synergistically with and performing part of the same function as the orbital region of the OOc. The decreased downwards pull on the orbital region of the OOc caused by the cheek raising by the Zmj could encourage resting tonus increases in the OOc, inducing a noticeable contraction. Zmj contraction also raises regions of the cheek over and adjacent to the orbital region of the OOc surrounding the eye. The sensation of malar tissue (the cheek) rising next to and over the orbital region of the OOc could also increase the probability of OOc contraction.

Another source could involve the motor neurons in facial nerve. The OOc typically receives inputs from the temporal and zygomatic branches of the facial nerve [9]. These branches are all extensively interconnected through a network known as the parotid plexus. The functions of these connections are unclear, but they are a potential source of the coordination between Zmj and OOc contraction that induces Duchenne smiling. Although fibers connecting the OOc and Zmj are not common, these potential sources might help in coordinating movements of the OOc and Zmj during a Duchenne smile.

### Interconnection of OOc, Zmi, and Zmj

Hur et al. (2018) [8] reported that muscle fibers extending from the OOc constituted the Zmi in all specimens. Kampan et al. (2018) [10] stated that the lateral bundles of the OOc joined with the Zmi in all specimens, and with the Zmj in 63.6% of 12 Japanese cadavers. In contrast, fibers connecting the OOc to the Zmj were observed in only 22.7% of specimens in the present study.

The Zmi elevates the upper lip, exposing the maxillary teeth. The main elevators of the lip (levator labii superioris alaeque nasi, levator labii superioris, and Zmi) act together to curl the upper lip for smiling and for expressing smugness, contempt, or disdain [11]. During these facial expressions, coordinated movements between the orbital region and upper lip (OOc and Zmi) therefore occur more often than between the orbital region and the corner of the mouth (OOc and Zmj).

### Consideration for facial cosmetic procedures

In the present study, the fibers connecting the OOc and Zmj coursed from the orbital region to the lower cheek and side of the mouth. Repeated contractions of the OOc and Zmj alongside the connecting fibers can therefore cause lower the crow’s feet and lateral cheek rhytides, forming accordion wrinkles. Accordion wrinkles form from the simultaneous action of two main muscles: the OOc and Zmj acting proximally and distally, respectively [12, 13].

## Conclusion

The results of this study provide further anatomical insight into the relationship between the OOc and zygomaticus muscle complex, which might be helpful for elucidating the anatomy of smiling and treatment and surgery designs for balanced or ideal smiles.

## Supporting information

S1 Fig Anatomical contribution of the orbicularis oculi (OOc) to the zygomaticus major (Zmj). (A) Superficial fibers of the orbital OOc extended directly to the Zmj. These muscle fibers (arrowheads) comprised the upper Zmj. (B) Superficial fibers of the orbital OOc extended to the zygomaticus minor (Zmi) and their small portion (arrowheads) joined to the upper Zmj fibers. (C) No connection was observed between the OOc and Zmj.
(TIF)
