# Supplementary figures and images for: Anatomical contribution of the orbicularis oculi to the zygomaticus major: An improved understanding of the smile with consideration for facial cosmetic procedures
Source: PLoS One. 2022 Jul 28;17(7):e0272060. doi: 10.1371/journal.pone.0272060 (PMC9333313; doi:10.1371/journal.pone.0272060)

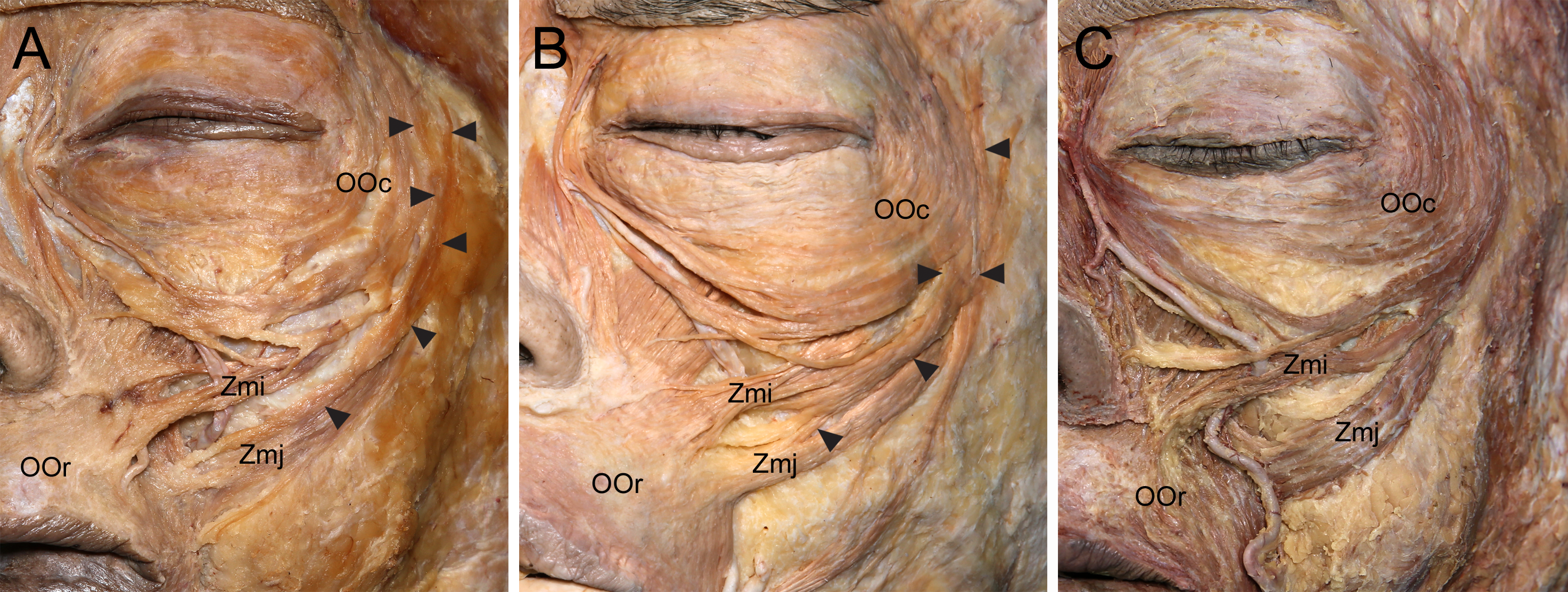

Supplement: S1 Fig — (A) Superficial fibers of the orbital OOc extended directly to the Zmj. These muscle fibers (arrowheads) comprised the upper Zmj. (B) Superficial fibers of the orbital OOc extended to the zygomaticus minor (Zmi) and their small portion (arrowheads) joined to the upper Zmj fibers. (C) No connection was observed between the OOc and Zmj. (TIF) [file pone.0272060.s001.tif]
